# There and back again; historical perspective and future directions for *Vaccinium* breeding and research studies

**DOI:** 10.1093/hr/uhac083

**Published:** 2022-04-11

**Authors:** Patrick P Edger, Massimo Iorizzo, Nahla V Bassil, Juliana Benevenuto, Luis Felipe V Ferrão, Lara Giongo, Kim Hummer, Lovely Mae F Lawas, Courtney P Leisner, Changying Li, Patricio R Munoz, Hamid Ashrafi, Amaya Atucha, Ebrahiem M Babiker, Elizabeth Canales, David Chagné, Lisa DeVetter, Mark Ehlenfeldt, Richard V Espley, Karina Gallardo, Catrin S Günther, Michael Hardigan, Amanda M Hulse-Kemp, MacKenzie Jacobs, Mary Ann Lila, Claire Luby, Dorrie Main, Molla F Mengist, Gregory L Owens, Penelope Perkins-Veazie, James Polashock, Marti Pottorff, Lisa J Rowland, Charles A Sims, Guo-qing Song, Jessica Spencer, Nicholi Vorsa, Alan E Yocca, Juan Zalapa

**Affiliations:** 1 Department of Horticulture, Michigan State University, East Lansing, MI, 48824, USA; 2 MSU AgBioResearch, Michigan State University, East Lansing, MI, 48824, USA; 3 Plants for Human Health Institute, North Carolina State University, Kannapolis, NC USA; 4 Department of Horticultural Science, North Carolina State University, Raleigh, NC USA; 5 USDA-ARS, National Clonal Germplasm Repository, Corvallis, OR 97333, USA; 6 Horticultural Sciences Department, University of Florida, Gainesville, FL 32611, USA; 7 Fondazione Edmund Mach - Research and Innovation Centre Italy; 8 Department of Biological Sciences, Auburn University, Auburn, AL 36849, USA; 9 Phenomics and Plant Robotics Center, College of Engineering, University of Georgia, Athens, USA; 10 Department of Horticulture, University of Wisconsin-Madison, Madison, WI, 53706, USA; 11 USDA-ARS Southern Horticultural Laboratory, Poplarville, MS 39470-0287, USA; 12 Department of Agricultural Economics, Mississippi State University, Mississippi State, MS 39762, USA; 13 The New Zealand Institute for Plant and Food Research Limited (PFR), Palmerston North, New Zealand; 14 Department of Horticulture, Washington State University Northwestern Washington Research and Extension Center, Mount Vernon, WA, 98221, USA; 15 SEBS, Plant Biology, Rutgers University, New Brunswick NJ 01019 USA; 16 School of Economic Sciences, Washington State University, Puyallup, WA 98371, USA; 17 USDA-ARS, Horticulture Crops Research Unit, Corvallis, OR 97333, USA; 18 USDA-ARS, Genomics and Bioinformatics Research Unit, Raleigh, NC 27695, USA; 19 Department of Crop and Soil Sciences, North Carolina State University, Raleigh, NC 27695, USA; 20 Department of Biochemistry and Molecular Biology, Michigan State University, East Lansing, MI, 48823, USA; 21 Department of Horticulture, Washington State University, Pullman, WA, 99163, USA; 22 Department of Biology, University of Victoria, BC, Canada; 23 USDA-ARS, Genetic Improvement of Fruits and Vegetables Laboratory, Beltsville, MD 20705, USA; 24 Food Science and Human Nutrition Department, University of Florida, Gainesville, FL 32611, USA; 25 Plant Biotechnology Resource and Outreach Center, Department of Horticulture, Michigan State University, East Lansing, MI 48824, USA; 26 Department of Plant Biology, Michigan State University, East Lansing, MI, 48824, USA; 27 USDA-ARS, VCRU, Department of Horticulture, University of Wisconsin-Madison, Madison, WI 53706, USA

## Abstract

The genus *Vaccinium* L. (Ericaceae) contains a wide diversity of culturally and economically important berry crop species. Consumer demand and scientific research in blueberry (*Vaccinium* spp.) and cranberry (*Vaccinium macrocarpon*) have increased worldwide over the crops’ relatively short domestication history (~100 years). Other species, including bilberry (*Vaccinium myrtillus*), lingonberry (*Vaccinium vitis-idaea*), and ohelo berry (*Vaccinium reticulatum)* are largely still harvested from the wild but with crop improvement efforts underway. Here, we present a review article on these *Vaccinium* berry crops on topics that span taxonomy to genetics and genomics to breeding. We highlight the accomplishments made thus far for each of these crops, along their journey from the wild, and propose research areas and questions that will require investments by the community over the coming decades to guide future crop improvement efforts. New tools and resources are needed to underpin the development of superior cultivars that are not only more resilient to various environmental stresses and higher yielding, but also produce fruit that continue to meet a variety of consumer preferences, including fruit quality and health related traits.

## Overview

Our goal for this article is to provide an overview of accomplishments and goals across a wide variety of research areas for fruit crops in the genus *Vaccinium*. Please note that this review does cover more heavily literature on blueberry and cranberry, only because previous studies have largely been conducted on these two crops relative to the other *Vaccinum* species. Thus, for many of the below sections, we will largely review what is known about blueberry and cranberry. We hope that this will emphasize the need for more comparative studies in *Vaccinium* that includes a wider diversity of wild and cultivated species. Nonetheless, we envision that this review will serve as a valuable resource for the broader *Vaccinium* research community and serve as a useful roadmap for future research.

## Systematics, diversity, and domestication history of the genus *Vaccinium*

The Ericaceae Juss., subfamily *Vaccinioideae* consists of five tribes, *Andromedae*, *Gaultherieae*, *Lyonieae*, *Oxydendreae*, and *Vaccinieae* [1]. *Vaccinieae* are morphologically diverse, spanning 33 genera and 1267 species, including the genus *Vaccinium* with 450 species [2]. Linnaeus first defined the genus *Vaccinium* in 1737 [3–10]. Vander Kloet (1981) [11] closely re-interpreted the Latin description, and redefined the lectotype to be the specimen of *Vaccinium uliginosum* first collected by Linnaeus. This type specimen is a terrestrial oxylophyte originating in the Northern Hemisphere with 4-meris flowers and eight stamens. *Vaccinium sensu lato* includes a broader definition: plants with continuous or divided corollas, 4-meris or 5-meris perianth, oxylophyte or calcicole, terrestrial plant, lithotroph, or epiphyte. Despite Linnaeus’s original description of representatives in the Northern Hemisphere, this genus encompasses diverse species from not only the Palearctic and Nearctic of the Northern Hemisphere, but the Indomalayan and Neotropical Realms of the Southern Hemisphere. This Linnaean definition set the stage for the polyphyletic challenges presently facing the genus. While this is a systematic challenge, polyphyletic groups might suggest unusual hybridization possibilities for breeders interested in broadening gene pools for cultivar development.

**Figure 1:**
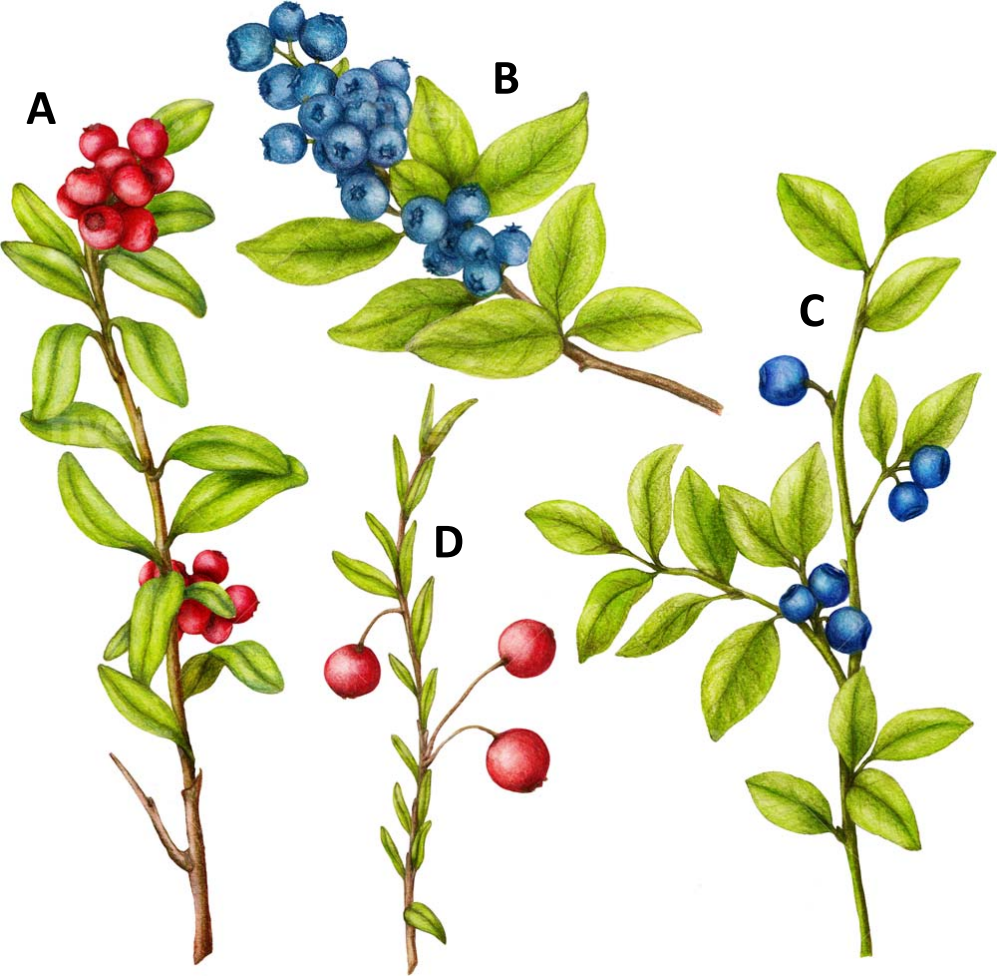
An illustration of A) lingonberry, B) blueberry, C) bilberry and D) cranberry by the artist Arevka.

Molecular studies have substantiated that the vast majority (60–80%) of described genera are highly polyphyletic, including *Vaccinium* [12, 13]. Thus, multi-marker molecular phylogenetic studies are needed to further resolve species relationships and guide taxonomic revisions for the genus *Vaccinium*, consisting of more than 450 described species [14, 15]. The main commerical crops are bilberry (*Vaccinium myrtillus* L.), cranberry (*Vaccinium macrocarpon* Aiton), lingonberry (*Vaccinium vitis-idaea* L.) and blueberries, which includes northern highbush blueberry (*Vaccinium corymbosum* L.), lowbush blueberry (*Vaccinium angustifolium* Aiton), and rabbiteye blueberry (*V. virgatum* Aiton [synonym = *V. ashei* J.M. Reade]) (Figure 1). Breeders are creatively crossing diverse species within different subgenera and are having surprising success obtaining viable offspring, but ploidy remains a common reproductive barrier [16]**.** Diploid (2x), tetraploid (4x) and hexaploid (6x) species have been described [17], with ploidy ranges discovered even within some species (e.g. diploid and tetraploid *V. corymbosum*; [18]). The majority of data, largely based on chromosome pairing behavior, suggests that these species are largely autopolyploids (i.e. three or more complete chromosome sets derived from a single progenitor species) [17]. However, the ancestry of these polypoid species remains pooly understood, thus, further supporting the need of additional taxonomic and phylogenetic studies across *Vaccinium*.

## Breeding priorities for blueberry, cranberry, and related crops

Blueberry and cranberry breeding through traditional methods is an expensive and time-consuming process, taking at least 10 years to release new cultivars [19, 20]. During the last three decades, significant resources in blueberry breeding were devoted to the development of low-chill southern highbush cultivars, which involved introgressing *Vaccinium darrowii* Camp into breeding lines [21]. The development of these new southern highbush cultivars contributed to the massive expansion of production into many non-traditional areas worldwide [21]. Besides chilling requirements, target breeding traits for blueberry improvement are yield, fruit quality (size, color, firmness, flavor, stem scar), vegetative characteristics (vigor, disease resistance, plant architecture), and adaptation (cold hardiness, self-fruitfulness, soil pH tolerance) [21]. For cranberry, stable year-to-year high yields is a critical trait as many cultivars exhibit a biennial bearing habit [20]. Other priority traits for cranberry improvement have been fruit quality (color, flavonoid content, sweetness, uniform large size), disease and pest resistance, and vegetative characteristics (vigor, adaptation including heat and cold tolerance) [20].

Accelerating the breeding process and combining multiple traits (pyramiding) using marker-assisted selection (MAS) are primary goals for both crops [19, 20]. To achieve this goal, between 2016–2018, as part of a planning grant funded by USDA-NIFA (Grant # 2016–51 181-25 401) [22], breeding priorities across the US blueberry and cranberry industry were assessed through a survey. The results of the survey, data from over 490 respondants, indicated that the development of cultivars with superior fruit quality traits, particularly with improved firmness, flavor, and shelf life was the top industry priority [23, 24]. Traits associated with machine harvestability and disease resistance were additional industry priorities for blueberry. Disease resistance and arthropod resistance were additional industry priorities for cranberry. Regional differences were observed especially for traits that depend on environmental factors (e.g. heat or cold tolerance).

The results of the survey reflected multiple challenges associated with *Vaccinium* spp. crop production and new regulations and market shifts that the industry is facing, all of which can be partially addressed by improving fruit quality through the use of various breeding techniques. A periodic re-evaluation of industry breeding priorities will be critical to ensure national and local scale research efforts are addressing industry needs. The identification of research priorities established a strong rationale for securing federal funds that can best serve the needs of the industry. For instance, it served as a foundation to establish the VacCAP project (https://www.vacciniumcap.org/), a multi-state and multi-disciplinary project whose mission is to advance genetic resources and develop new superior blueberry and cranberry cultivars.

## Breeding beyond the primary Genepool for blueberry

The primary genepool for blueberry consists of the commonly cultivated species; *V. corymbosum, V. angustifolium,* and *V. virgatum*. In the primary genepool, all species material originates in section *Cyanococcus*. While each blueberry of commerce is its own primary genepool, each is also a member of the secondary genepool for the other species. In a brief overview, below, are the primary blueberry species, and species that have been used in introgression. *Vaccinium corymbosum* L., the tetraploid version of the species, is the origin of highbush blueberry; it is upright, crown-forming, productive and self-fertile. *V. corymbosum* comprises approximately 90% of the ancestry of most standard northern highbush cultivars. *Vaccinnium angustifolium* Aiton is the semi-cultivated tetraploid species known in commerce as lowbush blueberry. It has value for early ripening and fruit quality. It is present in small amounts in most northern highbush cultivars, typically at percentages of 5–10%, and is present at percentages of ~25–50% in many half-high blueberry cultivars. Half-high cultivars are produced by hybridizing tetraploid *V. corymbosum* with *V. angustifolium*. *Vaccinium ashei* Reade (syn. *V. virgatum* Aiton) is a hexaploid species native to the southern U.S. *V. ashei* can introduce high vigor, and heat tolerance. Its negatives are late ripening and almost non-existent self-fruitfulness. Most (but not all) rabbiteye cultivars are 100% *V. ashei. V. ashei* is present in small percentages in many southern highbush cultivars. Southern highbush cultivars have been developed by introgressing *V. darrowii* into tetraploid *V. corymbosum.*

In the secondary genepool for blueberry, the introduced species material originates in section *Cyanococcus*, the same taxonomic section as the primary genepool; however, these materials have had few efforts at domestication and are essentially wild. Nonetheless, a number of secondary genepool species have successfully been used in breeding. To date the largest introgression of germplasm beyond the primary genepool has been the use of *V. darrowii* for the development of southern highbush blueberry. *V. darrowii* is a southern diploid species with a colonial habit, glacous foliage, and no chilling requirement. It was used in the development of southern highbush (SHB) with the primary goal of introducing adaptation for lower chill requirements for bloom. Several additional, valuable traits were also recognized in this material, notably vigor, unique fruit volatiles, fruit firmness, and waxy foliage. Introgression of *V. darrowii* into a northern highbush background is virtually a defining aspect of the many southern highbush cultivars available. *V. darrowii* is typically present at a level of about 25%. *Vaccinium elliottii* Chapm. has the potential to introduce vigor and early flowering. *V. elliottii* has been successfully used in three commercial cultivars: “Carteret” (SHB 25%), “Snowchaser” (SHB 19%)*,* and “Kestrel” (SHB 6%). *Vaccinium constablaei* Gray is a hexaploid high-altitude species. Because of this, it can introduce the characteristics of late bloom, compact bloom to ripe time, earlier ripening, and extreme cold hardiness. It also has fruit composition comparable to highbush. Three cultivars contain *V. constablaei* germplasm: “Sierra” (SHB), “Cara’s Choice” (SHB), and “Snowflake” (RE). *Vaccinium tenellum* Aiton is reputed to be drought tolerant, and some selections have been noted as having high numbers of contiguous buds (Ehlenfeldt, personal observation). It also has the highest recorded number of volatile aromatic components [25]. *V. tenellum* is present in four cultivars: “Bladen” (SHB), “Reveille” (SHB), “Sweetheart” (HB-SHB), and “Pink Lemonade” (RE-Mixed species).

The tertiary genepool crosses for blueberry are intersectional crosses. A number of modern efforts have focused on thus far unused or unincorporated species of this genepool. Thus far no cultivars have been released from such crosses, but the promise of these materials is high. *Vaccinium stamineum* L. (section *Polycodium*) is a native diploid southern specie s with the potential to introduce drought tolerance as well as unique fruit volatiles. Lyrene (2016) used colchicine-doubled species selections as males with numerous highbush blueberry clones as females to produce several hundred 4x hybrids [26]. These hybrids were shown to be fertile with 4x highbush blueberry. *Vaccinium arboreum* Marshall (section *Batodendron*) is a tree-like, diploid, southern species. Its value lies in its adaptation to upland soils and monopodial structure that may be amenable to machine harvesting. Lyrene (2011) again used colchicine-doubled species selections as males with numerous highbush blueberry clones as females to produce more than 1500 hybrids [27]. Subsequent studies demonstrated the fertility of these hybrids in backcrosses to highbush blueberry [26]. *Vaccinium padifolium* J.E. Sm. ex A.Rees (section *Hemimyrtillus*) is a tetraploid species native to the Azores. Its characters of value include profuse flowering, reflowering, and high self- fertility. Ehlenfeldt and Polashock (2014) produced several highly fertile hybrids with 4*x V. corymbosum* [28], and evaluated BC_1_ progeny under field conditions [29]. These workers have also produced putative hybrids with two other section *Hemimyrtillus* species, *V. cylindraceum* and *V. arctostaphylos* (Ehlenfeldt, personal communication). *Vaccinium bracteatum* Thunb. (section *Bracteata*) is a diploid species native to southeast Asia. There is substantia interest in this species for its pigmented flesh and the potential to introduce pigmented flesh and high antioxidants to commercial blueberry. It also possesses tolerance to higher pH soils. Tsuda et al. (2013) using a colchicine-doubled species selection [30], produced 66 4*x* hybrids and some hybrids were shown to have pigmented flesh a subsequent manuscript Tsuda et al. (2014) documented the superior rooting of several hybrids at higher pH levels [31]. *Vaccinium meridionale* Swartz is a tetraploid native to Jamaica and Colombia. *Vaccinnium meridionale* is of interest for its profuse flowering, concentrated flowering, panicle-like inflorescence structure, monopodial plant structure, and potential frost tolerance. Ehlenfeldt and Luteyn (2021) demonstrated production of fertile 4x hybrids [16], and Ehlenfeldt and co-workers (2018) have offered a preliminary report that *V. meridionale* can also cross with cranberry and lingonberry [32].

## Breeding beyond the primary Genepool for Lingnonberry

Unlike highbush blueberry, lingonberry (*V. vitis-idaea* L.) has a relatively limited accessible genepool. The introgression of genetic diversity from other species into lingonberry breeding programs has the potential to improve a wide variety of important target traits, including disease resistances, abiotic stress tolerances and a novel combination of fruit quality characteristics (e.g. flavor profiles). There are two recognized regional varieties or subspecies: 2x *V. vitis-idaea* subsp. *vitis-idaea* found in Eurasia, colloquially termed cowberry. This sub-species is the major lingonberry of commerce. The other sub-species found in North America is 2x *V. vitis-idaea* subsp. *minus* (Lodd.). For lingonberry, most breeding has focused on broadening the collected diversity of the primary gene pool [33]. There are however many indications that lingonberry can transgress its primary gene pool. Spontaneous natural hybrids between 2x *V. vitis-idaea* (cowberry) and 2x *V. myrtillus* (bilberry), termed *Vaccinium × intermedium* Ruthe, are occasionally found in Europe and were extensively investigated by Ritchie (1955) [34, 35]. These hybrids although slightly fertile have not led to any major introgressions into commercial lingonberry. Due to morphological similarities, crosses of 2x *V. vitis-idaea* (lingonberry) and 2x *V. macrocarpon* (cranberry) were attempted, and readily produced hybrids, but were limited by near complete sterility [36, 37] . More recently Zeldin and McCown (1996) produced intersectional hybrids of the Hawaiian species 2x *Vaccinium reticulatum* section *Macropelma (Ōhelo berry)* x 2x *V. vitis-idaea*, section *Vitis-idaea* (lingonberry) [38]. Only limited fertility was reported to exist. The most promising prospects for truly broadening the genepool of lingonberry comes from more recent studies. In Belarus, Morosov (2007) succeeded in crosses between 4x *V. uliginosum* (bog bilberry) and a local 4x population of lingonberry [37]. These F_1_ hybrids possessed fertility and were successful in subsequent crosses as a female with colchicine-generated 4*x* cranberry, 4*x V. corymbosum* (highbush blueberry), 4*x V. corymbosum* - *V. angustifolium* (half-high blueberry), and 4*x* lingonberry. Morosov (2007) also reported successful crosses of 4*x* lingonberry × 4*x V. angustifolium* (lowbush blueberry) [37]. Some of these materials have been generationally advanced by Marozau and Baranov (2018) who describe trispecific hybrid / hybrids of (*V. uliginosum* L. × *V. vitis-idaea* L.) × *Oxycoccus macrocarpus* (Aiton) Pursh (aka *V. macrocarpon*/cranberry) [39]. Ehlenfeldt and co-workers (2018) have similarly demonstrated highly fertile 4x F_1_hybrids with lingonberry originating from crosses of 4x *V. meridionale* (Andean blueberry) and 2x *V. vitis-idaea* (arising from 2*n* gamete function in *V. vitis-idaea*). These hybrids show indications of significant self-fertility, and good cross-fertility with *V. vitis-idaea*, 4*x V. corymbosum*, and 4x *V. macrocarpon*.

## Breeding beyond the primary Genepool for cranberry

Much like lingonberry, the genepool of cranberry is limited. The section *Oxycoccos* contains only two species: *Vaccinium oxycoccos*, the common cranberry, a wild diploid found throughout the cool temperate Northern Hemisphere, and *V. macrocarpon*, the diploid large cranberry or American cranberry. Among the earliest wide crosses in cranberry development are the previously mentioned hybrids of cranberry and lingonberry [36, 37]. Zeldin and McCown (1996) produced the aforementioned intersectional hybrids of 2x *V. reticulatum* section *Macropelma (Ōhelo berry)* × 2x *V. macrocarpon*, section *Oxycoccos* (cranberry) [38]. Like their crosses between *V. reticulatum* and lingonberry, very limited fertility was reported to exist. Vorsa et al. (2008) generated a blueberry × cranberry hybrid from an intersectional cross of 2*x V. darrowii* (Darrow’s blueberry) × 2x (*V. macrocarpon* × *V. oxycoccos*) [40]. These hybrids were diploid, and showed only limited vigor and fertility. Some plants have been advanced through subsequent crossing cycles. Recently significant success has been achieved by Ehlenfeldt and co-workers (2018) producing fertile 4x F_1_ hybrids from crosses of 4*x V. meridionale* (Andean blueberry) and 4*x V. macrocarpon* (cranberry)(ploidy variant research clones) [32]. This cross was easily accomplished, and over 500 hybrids were produced in the first crossing cycle. These hybrids have shown significant vigor, intermediate morphology, indications of fertility at least among some clones, and sufficient variation in hybrid morphology to expect useful selection and recombination for morphological traits (Ehlenfeldt, personal communication). It is apparent that the secondary and even tertiary gene pools are valuable resources that could be utilized to improve a wide variety of key target traits, including disease resistances, abiotic stress tolerances and fruit quality characteristics (e.g. sugar content). The use of this diverse germplasm should allow improvements in commercial blueberry, lingonberry, and cranberry crops, and may ultimately generate morphologically intermediate commercial crops yet to be visualized.

## Genomic, transcriptomic, and metabolomic resources for *Vaccinium*

While there have been great strides in blueberry cultivar development, genomic resources are needed to expedite this process to meet consumers’ increased demand and changing preferences. There has been significant development of marker-assisted breeding resources (further discussed in a subsequent section), but next-generation genomic, transcriptomic and metabolomic resources are critical to guide the development of markers. Publicly available “omic” resources for *Vaccinium* species are described below, as well as suggestions for future resources needed for the *Vaccinium* community.

Ploidy levels across *Vaccinium* spp. vary from diploid to hexaploid [41, 42]. The haploid (*n* = 12) genome size is estimated to be ~608 Mb [43]. A draft genome of diploid *V. corymbosum* (accession “W85–20”) was the first to be assembled and annotated [41, 44]. This assembly represented about ~358 Mb and consisted of 15 129 scaffolds, and has served as a valuable resource for mining genes for disease and quality improvement [45]. An improved tetraploid blueberry genome assembly (2*n* = 4*x* = 48) was completed by Colle et al. (2019) for the northern highbush cultivar Draper (*V. corymbosum*) using a combination of different sequencing technologies [46]. This chromosome-scale genome consists of 48 pseudomolecules spanning ~1.657 Gb. Genome annotation produced 32 140 protein-coding genes per haplotype and analysis of the genome addresses the likely allopolyploid origins of tetraploid highbush blueberry [46]. Gene expression analysis was also performed to identify key candidate genes associated with fruit metabolites. This genome assembly serves as an invaluable resource for guiding current molecuar breeding efforts, but also for evolutionary studies within the order Ericales.

The first American cranberry (*V. macrocarpon*) draft genome (2*n* = 2*x* = 24) was assembled by Polashock et al. (2014) using short-read Illumina technology [47]. A 5^th^ generation inbred genotype (CNJ99–125-1) was used for genome sequencing to reduce heterozygosity of the genome. The cranberry genome was estimated to be ~470 Mb and ~ 420 Mb was assembled into 229 745 scaffolds. This assembly predicted 36 664 genes with 35% having transcriptome evidence supporting the exons. An improved chromosome-scale genome assembly of cranberry was released in 2021 (*V. macrocarpon* cv. Stevens) along with a draft genome assembly of bog or small cranberry (*Vaccinium microcarpum*), a close wild relative of *V. macrocarpon* [48]. The updated cranberry draft genome contains more than 92% of the estimated 492 Mb genome in 12 chromosomes and genome annotation predicted 23 523 protein-encoding genes. The draft *V. microcarpum* genome has a total length of 622 Mb in 4802 scaffolds and contains 30 147 predicted protein-encoding genes. A genome of a 5th generation inbred genotype (CNJ99-125-1) of cranberry was recently published, as well as a reference-quality genome of small-fruited cranberry (*V. oxycoccos*) ([199]). Estimated genome sizes were 487 Mb and 585 Mb respectively, with BUSCO scores of 95 and 94 respectively. Together, these genomes enable the investigation of polyploidization in the Ericaceae, anthocyanin production in cranberry fruit, phytonutrients and other traits and serve as a resource for future breeding efforts.

Recently, a draft genome assembly of bilberry (*V. myrtillus*) has also been made publicly available [49]. Long-read sequencing was completed on *V. myrtillus* (isolate NK2018) producing a 524 Mb genome assembly in 1418 scaffolds. The genome of the undomesticated bilberry will provide a useful comparison with domesticated *Vaccinium* spp., particularly for understanding complex traits such as anthocyanin regulation and distribution. Another recently sequenced species is *V. darrowii* (evergreen blueberry) [50]. Pacific Biosciences Circular Consensus long-read Sequencing (CCS) was used to develop a reference quality 1.06 Gb (2*n* = 2*x* = 24) genome assembly comprised of 491 scaffolds (107 primary and 384 secondary haplotype sequences) with over 97.8% of the assembly scaffolded into 24 pseudomolecules [50]. Transcriptome sequencing across berry development allowed for annotation of a total of 64 526 genes across both haplotypes. Although no genome is available for *V. vitis-idaea* (lingonberry), transcriptome sequence identified 67 836 genes including many differentially expressed during fruit development [51]. The Genome Database for Vaccinium (GDV, https://www.vaccinium.org) [52], the crop community database for *Vaccinium*, provides access to integrated genomic, genetic, and breeding peer-reviewed published data, and analysis tools. The repository in GDV contains gene, genome, genetic map, marker, phenotype, publications, qtl, species, transcriptome, and trait data curated by the GDV team. Tools include a genetic map viewer, genome browser, synteny viewer, metabolite pathways browser, sequence retrieval, BLAST, and breeding information management system (BIMS). A suite of search tools provides intuitive querying of all major data types. Soon, additional *Vaccinium* genomic resources will be available through the Vaccinium Coordinated Agricultural Project (VacCAP; USDA Award No: 2019–51 181-30 015 [53]). VacCAP is a US nation-wide transdisciplinary project to improve blueberry and cranberry by developing and implementing marker assisted breeding [53] and will enable effective association mapping studies in blueberry and cranberry.

A key focus of the VacCAP project is the development of a *Vaccinium* pangenome. A pangenome is a collection of genetic material present within a taxonomic group [54]. Dozens of plant pangenome studies have proved critical in the understanding of plant genome biology [55]. These studies classify genes as core or dispensable. Core genes are those present in each individual genome while dispensable genes are absent in at least a single individual. Consistently, core gene functions are enriched for routine metabolic functions while dispensable gene functions are enriched with stress responsive and specialized metabolism. The regions encoding variation of several key target traits of interest likely lie in the dispensable portion of the genome. Since dispensable portions are absent in at least a single individual, a proportion of them will be absent in any single reference genome. Therefore, the construction of a pangenome is required to identify and characterize all important genes, and to develop markers linked to them to guide future breeding efforts.

Next-generation sequencing technologies also provide an unprecedented opportunity to study genome-wide transcript expression levels through RNA-sequencing and transcriptomics. Initial expression studies focused on chilling unit accumulation, vernalization, flower bud development, fruit development and quality traits, and cold acclimation [56]. The recent availability of high-quality reference genomes for *Vaccinium* (blueberry, cranberry, bilberry) and technological advances in RNA-sequencing have also advanced transcriptome profiling. Recent transcriptome studies in *Vaccinium* have focused on the biosynthesis of metabolites with human health benefits [44, 46, 51, 57–65], cold acclimation [66–71], fruit and flower development [72–77], biotic interactions [78–82], postharvest storage [83, 84], roots [85, 86], and transcriptome regulatory analysis [87–89]. As of June 2021, there are 543 entries for “*Vaccinium*” in the NCBI Small Read Archive (SRA), encompassing varying species, developmental stages, sequencing technologies, tissue type, and sequencing strategy. Transcriptome and transcript expression data can also be found through GDV which is a curated web-based database [52], and future resources will be made available through VacCAP. More work is needed to expand the transcriptomic resources for other *Vaccinium* spp*.*, as well as abiotic stress-based research to understand the impacts of changing growing conditions on fruit and nutritional quality for future breeding efforts.

While databases for genome and transcriptome data are available, there is no central and comprehensive resource to access metabolic information specific to *Vaccinium* spp. Metabolomics databases typically contain datasets of spectra, structure, annotation, or pathways, but only a few databases include information on the biological sources of metabolites. The KNApSAcK database [90, 91] has 56 829 metabolites and 135 156 metabolite-species pairs (updated April 8, 2021), of which 99 metabolites are associated with *Vaccinium* spp. (i.e. input type = all, input word = Vaccinium) with a total of 146 metabolite-species relationship (i.e. input type = organism). MetaboLights [92] also has a species-search function, but very few results are retrieved using “Vaccinium” as a search term. FooDB [93], a resource for nutrient information of various foods, can be used to search by food name or scientific name and currently lists 20 entries for *Vaccinium*. The Food Metabolome Repository [94, 95] contains raw metabolome data of supermarket-purchased blueberries analyzed by liquid chromatography-mass spectrometry (LC–MS) and includes information on compound peaks; however, users should be cautious of the possible false-positive and false-negative results due to non-replication of sample preparation. Nevertheless, a number of papers reporting metabolite composition of several *Vaccinium* spp. have been published, including comparative studies (e.g. among species, across developmental stages) and assessment of responses to different environmental conditions [96–98]. Since *Vaccinium* spp. are known for their human health benefits attributed to the presence of bioactive compounds [99], most studies focus on secondary metabolites such as phenolic compounds and other antioxidants that contribute to fruit quality. Aside from fruits, metabolite profiles of other plant tissues such as leaves, roots, flowers, and stems have also been characterized. Supplementary Table 1 summarizes recent reports on primary and secondary metabolites found in different organs of wild and cultivated *Vaccinium* spp. at different developmental stages and under various experimental conditions.

With the increasing use of metabolomic techniques, more studies on the metabolite composition of *Vaccinium* spp. are expected. As depositing data in a metabolomics repository becomes a requirement for publishing, this will allow for more comprehensive metabolite resources to be available and easily accessible in the future. While general plant metabolite databases are available for metabolite identification, species-specific differences could complicate data analysis for *Vaccinium*. For example, a recently constructed tomato-specific metabolome database contains accurate mass records with 71% mass values not found in mass records of *Arabidopsis thaliana*, *Medicago truncatula*, or *Jatropha curcas* [100]. Hence, a metabolome database dedicated to *Vaccinium* spp. is worth developing.

A repository of *Vaccinium* metabolite data generated by a consortium of laboratories will be crucial for establishing metabolomics as a functional genetics tool. To gain a more holistic understanding of how gene-metabolite networks determine specific traits, data integration of two or more ‘omics’ datasets is essential. Two basic strategies are commonly described for linking ‘multi-omics’ datasets; the ‘top down’ data reduction approach which employs global transcriptomics and/or genomic data to predict phenotypic and/or metabolic responses and the ‘bottom-up’ approach, which uses metabolomic data to target upstream transcriptomic/proteomic processes for the discovery of mechanistic genes [101]. The recent emergence of ‘multi-omics’ data analysis and integration tools, such as MixOmics [102], MetaboAnalyst [103] and OmicsAnalyzer [104], have been crucial in facilitating data integration from post-analysis approaches, synthesizing individual ‘omics’ analyses, to merging ‘multi-omics’ data sets for simultaneous supervised or unsupervised analyses. Recent studies using target ‘bottom up’ data-integration approaches have proven powerful for identifying candidate genes regulating blueberry secondary metabolites affecting fruit flavor [105] and skin color [58]. Despite recent advances in genomic resources for *Vaccinium* untargeted “top-down” or systems modeling approaches, combining large-scale biological datasets, are yet to be explored. A prerequisite for such analyses is publicly available comprehensive genomic, transcriptomic, metabolomic and/ or proteomic datasets in addition to a community-based platform for connecting such data. The recently established Paired Omics Data Platform [106] is an exciting initiative to help streamline access to paired genomic and metabolomic data from public databases and repositories. The use of such resources will rapidly extend our knowledge beyond single candidate genes to build interaction networks from ‘multi-omics’ data that can be interrogated to identify genomic features that affect *Vaccinium* spp. phenotypes.

## Genetics of fruit quality and related target traits

Fruit quality is a term that may convey several interpretations when applied to fruits and vegetables depending on its intended use and to the segment of the production chain applied. For example, at the base of the production chain are the growers, to whom quality mostly corresponds to berry size, firmness, color, absence from defects, and economic potential for the intended market. In another segment of the production chain are the retailers for whom quality is primarily associated with postharvest attributes, including appearance, uniformity, and the shelf life of the product. Lastly, at the top of this hierarchy are the consumers who are seeking premium appearance, certain sensory attributes (e.g. texture, flavor), and nutritional value. These differences in priorities in the production chain have created new challenges to modern blueberry and cranberry breeding programs, to release new cultivars that produce higher quality fruit without jeopardizing the horticultural traits required by growers and wholesalers. In this regard, Beaudry (1992) suggested a set of minimum criteria for quality standards considering some important traits in blueberry (Table 1) [107]. After two decades of breeding, we revised some of these values established in the 1990’s and noticed that the rapid genetic progress has changed the standards for fruit quality (Table 1).

**Table 1:** Quality standards recommended for blueberry fruit originally reported by Beaudry (1992) [107] and adapted by Retamales and Hancock (2018) [122] with the range heritability (*h^2^*) values compiled from multiple blueberry studies [107, 122]

**Attribute**	**Original standards** [ [107]]	**New standard** [122]	***h*** ^***2***^	**References for the *h*** ^***2***^ **values**
pH	2.25–4.25	3–4	0.35–0.50	[109, 112, 113]
SS	>10%	> 11%	0.35–0.65	[109, 112, 113]
SS:TTA	10–33	15–30		
Firmness	>70 g	>200 g	0.40–0.70	[109, 112, 114]
Size	>10 mm	>15 mm	0.14–0.65	[109, 112, 114]
Color	Blue	Blue	0.80	[114]
Aroma	*to be defined*		0.50–0.80	[105]
Antioxidant			0.43	[120]
Total Phenolic			0.46–0.70	[113, 120]
Anthocyanin			0.45–0.80	[113, 120]
Flavanal			0.15–0.70	[113]
Flavonol			0.15–0.50	[113]
Phenolic acid			0.30–0.70	[113]

Among the primary fruit characteristics, firmness is one of the traits that has experienced the fastest breeding progress in blueberry. As an important proxy for texture quality, firmness is highly appreciated by both growers and consumers and is important for machine harvesting, contributing to the reduction of internal bruising and delaying subsequent postharvest decay. In a comprehensive review, [108] reported a large diversity between and within blueberry types and most importantly, a substantial improvement in modern cultivars compared to the first-released cultivars [110]. The increment of more than 250% in the current quality standard (Table 1) is not only an indication of intense breeding progress, but also highlights the fast response to selection for the texture trait. To support this, Cellon et al. (2018) reported a large narrow-sense heritability for firmness (*h^2^* > 0.70 for evaluations conducted in 2015 for SHB), while [110] and Qi et al., (2021) reported different quantitative trait loci (QTLs) for texture via association analyses [109–111].

Fruit size is another trait that has experienced profound changes during the breeding history of blueberries. A large variability among cultivars and wild species has been reported [112]. Inheritance of fruit size was recently studied in diverse breeding populations and presented a medium-to-large narrow-sense heritability [109, 112, 113]. In a recent study, Mengist et al. (2020) reported that fruit size can be estimated from fruit weight or volume and vice versa [113]. Seven significant associations were reported for fruit size in a genome-wide association (GWA) study [110]. Interestingly, an significant association for firmness and size co-localized in both a QTL and GWAS study, indicating good prospects for simultaneous selection for both traits [111].

Sugar and acids are another set of attributes commonly reported in *Vaccinium* affecting sensory perception and therefore consumer preferences. An adequate sugar-acid balance is important for blueberry flavor perception. Beaudry (1992) reported a soluble solids (SS) and total titratable acidity (TTA) ratio ranging from 10 to 33 as adequate [107]. However, new cultivars have a narrower SS/TTA ratio interval (15–30). This is evident when older and modern blueberry cultivars are compared, which shows a trend of reduced SS/TTA ratios over the past decades (Gündüz et al., 2015). From the genetic standpoint, the heritability of SS has been assessed in different studies with values ranging from 0.3–0.68 in different breeding populations [109, 112, 114]. In contrast, acidity has been measured in terms of pH and TTA with lower heritability values [109, 112, 113]. While for SS, only minor QTL were reported in both GWAS and QTL mapping studies [110, 111], a single major QTL was recently reported for pH [115].

Yield is a classic breeding target and has been measured in blueberry using different metrics such as grams per plant, kilograms per hectare, visual scores, or via yield-associated traits. Yield is polygenic and highly influenced by environmental factors, with low-to-medium narrow sense heritability values being reported for different populations and blueberry species (*h^2^* = 0.30–0.58), without any significant major QTL reported [109, 110, 116]. A valid alternative to traditional yield assessments in blueberries is through the use of secondary traits or via indirect selection. For example, a collection of fruit set traits with high heritability was recently assayed [111, 114].

From a quantitative genetics standpoint, most of the traditional traits associated with fruit quality are under polygenic control and display continuous phenotypic expression, moderate heritability values, and are largely influenced by environmental conditions [108, 109, 114, 117, 118]. Regarding gene action, there are few studies on the importance of additive and dominance effects at the mean phenotypic performances using proper genetic designs. Finn and Luby (1992) reported the use of a partial diallel mating scheme with a mixture of species and showed significant general combining ability (GCA) effects for color, picking scar and firmness traits in blueberry [119]. However, no significant variation for specific combining ability (SCA) was observed, which suggests greater importance to additive over dominance gene actions. In contrast, Bell et al. (2010) reported significant SCA effects for fruit set traits in lowbush blueberries [116], which suggests that non-additive effects may play an important role in traits associated with productivity.

More recently a second group of metabolite traits has been explored at the breeding level, with the potential for flavor and nutritional improvements. In sharp contrast to all previous traits, there is empirical evidence suggesting that such metabolites have a relatively more simple genetic architecture controlled by a few major QTL across the genome. For example, in blueberries, large values of genomic and pedigree-based heritability (*h^2^* > 0.5) were reported for a group of eleven volatile organic acids (VOCs) related to flavor preferences [105]. For bioactive compounds, Connor et al. (2002) and Mengist et al. (2020) also reported large heritability values for anthocyanin, flavonal, flavonol, and phenolic acid components [113, 120]. To emphasize the importance of such traits for breeding, a recent consumer perception survey showed that consumers are most interested in fruit aspects related to flavor and human health attributes [121]. The challenge ahead is to understand the metabolite diversity, their relationship to consumer preferences, disease resistance, and other associations, and then define tangible criteria for future breeding.

Besides the recent advances in blueberry and cranberry genetics, our understanding of the genetic mechanisms controlling various traits in blueberry and cranberry is still limited. Few QTLs have been validated across multiple studies and very few candidate genes have been identified. The increasing availability of genetic and genomic tools for *Vaccinium* crops promises an opportunity to close these gaps in the near future. For example, multiple genetic studies targeting several fruit characteristics such as harvest and postharvest studies to assess texture and storage index, organic acids and metabolites are currently ongoing as part of the VacCAP project. Along with other projects, these efforts will establish a roadmap for developing and implementing a marker assisted breeding (MAB) strategy in *Vaccinium* spp. crops.

## Molecular markers and high-throughput genotyping platforms

Diverse tools and approaches have been used to guide breeding efforts in *Vaccinium* spp. Dominant markers, including random amplified polymorphic DNA (RAPD) and amplified fragment length polymorphism (AFLP), were developed and used in blueberry and cranberry [20, 123]. Species-specific sequence-characterized amplified region (SCAR) markers which can be both dominant and codominant, were developed for rapid genotyping of cranberry [124]. Co-dominant molecular markers such as simple sequence repeat (SSR) and expressed-sequence tag-polymerase chain reaction (EST-PCR) markers became first available in *V. corymbosum* L. in the early 2000s [125–128]. Transferability of some of these SSR markers [125] enabled their use in other *Vaccinium* species such as in section *Cyanococcus* [129] and in other sections like *Oxycoccus* in cranberry (*V. macrocarpon*) [130]. In blueberry and cranberry, SSRs were extensively used to assess relatedness and genetic diversity [41, 126, 131–133], for cultivar identification [134–136], and linkage mapping [137–140]. As Next Generation Sequencing (NGS) technologies were applied in *Vaccinium*, species-specific SSRs were developed (e.g. *V. floribundum* [141]; *V. macrocarpon* [136]), and the era of high throughput genotyping and single nucleotide polymorphism (SNP) markers began in blueberry and cranberry.

In tetraploid cultivated blueberry (*V. corymbosum*), both non-targeted [e.g. genotyping by sequencing (GBS) or double-digest restriction site-associated DNA sequencing (ddRAD-seq)] and targeted (e.g. sequencing of amplicon or baited target capture) approaches to high throughput genotyping are in use, whereas, in cranberry only GBS has been utilized thus far. GBS has been used in blueberry for the analysis of genetic diversity [142, 143], linkage mapping [144–146], QTL analysis [144, 145], and to validate F1 hybrids of *V. padifolium* x *V. corymbosum* [16]. Genome-wide SNP data identified via ddRAD-seq indicated admixture in southern highbush blueberry and suggested a polygenic adaptation to low chill conditions [147]. In cranberry, GBS has been extensively used for linkage mapping [140, 148, 149]. The high-density linkage maps corresponded to the expected 12 chromosomes and allowed the identification of many cranberry QTLs associated with fruit rot resistance [140], fruit shape and size-related traits [150, 151], fruit color, total anthocyanin content (TAcy), and Brix [152]. Cranberry GBS data have also been used in multivariate genomic best linear unbiased prediction (GBLUP) approaches to test the accuracy of genomic selection for fruit weight and yield [153]. Genome-wide sequence variation at 21000 GBS SNP loci in a diverse historical cranberry collection uncovered a gradual reduction of wild alleles as cranberry was domesticated [154]. GWAS in this diverse panel identified marker-trait associations (MTA) for average fruit weight and fruit rot but not for other traits, likely due to recurrent introgression of wild relatives and limited recombination events during cranberry domestication [154].

While GBS is an attractive and relatively economical method for generating large datasets, it suffers from a lack of reproducibility, high error rates and a high number of missing data points. Furthermore, the sequencing read depth obtained using GBS is often highly variable between loci and samples, making it very challenging to accurately estimate allele-dosage. Therefore, while GBS has been an efficient method for genotyping diploid cranberry, it has only limited application for polyploid *Vaccinium* spp. such as highbush and rabbiteye blueberries. The target capture method is also sequence-based genotyping; however, the method is more reproducible as it targets a specific set of probes across the genome. Nevertheless, unlike SNP arrays that target a fixed set of SNPs and are therefore biased toward the material used for SNP detection and selection, target capture enables the discovery of *de novo* SNPs as well as large-scale genotyping. Target capture also generates read depths that are more consistent between samples and loci, which facilitates sequencing experiments and the use of appropriate read depths to correctly estimate allele dosage. Ferrao et al. (2018) first demonstrated some of the advantages of dosage calling or tetraploid marker calling for blueberry genotypes generated via target capture for GWAS [110]. Dosage calling identified 15 SNPs associated with five fruit-related traits while the diploid model generated seven SNPs that were associated with two traits [110]. This approach was also used for constructing the highest-density linkage map in diploid [111] and tetraploid [14, 105] blueberries. SNP loci significantly associated with fruit quality traits, chilling requirements, and cold hardiness were identified in diploid blueberry [111], and with fruit firmness [144] and volatile organic compounds controlling fruit flavor in the tetraploid blueberry [105]. Target sequencing also has been used to genotype a diversity panel of 280 blueberry accessions and cultivars collected from NC State breeding program and NCGR, Corvallis. A GWAS analysis was carried out on 24 phenotyping traits including phenology of the plants and fruit quality related traits (H. Ashrafi, unpublished).

While GBS and target capture are economic and scalable (i.e. producing thousands of loci for thousands of samples), they both require bioinformatics capability and robust reproducible workflows for data analysis. Development of such high-throughput genotyping platforms, which are goals of Breeding Insight [155] and VacCAP, [53] along with the concurrent improvement of software and reproducible workflows/pipelines that can automate dosage calling and training of the workforce in their uses (“Tools for Polyploids”, [156]) will accelerate the rate of MTA discovery, facilitate GWAS and genomic selection, and enable marker-based selection and the development of new superior cultivars.

Whole-genome sequencing is the superior approach to assess genetic variation within a species, germplasm set or MTA in populations. Given that the cost of sequencing keeps decreasing, it is likely that whole-genome re-sequencing will be used in the future for genotyping individuals using SNPs, instead of the current genotyping methods such as SSRs and GBS/target capture. Beyond this, long-read sequencing will allow for high-throughput characterization of genome structural variation poorly captured by all current methods.

## Genome-assisted breeding in *Vaccinium*

Despite the success and great contribution that conventional phenotype-based selection methods have made for the blueberry and cranberry industries, modern genomic tools have shown the potential to reshape current breeding programs by accelerating breeding cycles and increasing genetic gains. By assessing the genome-wide variability and, consequently, the genetic makeup of individual plants, genomic-assisted breeding methods can facilitate the genetic dissection of complex traits, increase the precision and efficiency of selection, reduce the time and field trials necessary for cultivar development, and assist in the maintenance of the genetic diversity in a breeding population. These techniques are especially advantageous for perennial species, such as blueberry and cranberry, given the polyploidy in blueberry, heterozygosity, and long generation time to evaluate certain traits in mature plants. Moreover, they are useful to predict traits that are difficult, expensive, time-consuming and/or destructive.

Genomic tools comprise several techniques that require varying marker densities, breeding populations, and statistical foundations. Parallel to the advancements and declining costs of sequencing technologies for providing high-quality reference genomes and millions of SNP markers, the recent development of computational and statistical methods for genomic data manipulation, especially for polyploids, were key milestones for making genomics-assisted breeding a reality in the blueberry breeding community. Here, we will present recent progress in terms of genomics-assisted breeding using the following methods: linkage map, QTL mapping, GWAS, and genomic selection (GS).

Linkage maps provide the framework for haplotype inference and detection of major QTLs in association analyses. Furthermore, they can be used as a scaffolding strategy to increase the contiguity of genome assemblies and to provide insights into meiotic behavior, recombination rate variation, chromosomal rearrangements, and genome evolution [157]. Following the advance of genotyping platforms, genetic linkage maps in blueberry have become continually more saturated and higher resolution by including more markers and recombinant individuals, respectively. Moreover, new methods addressing autopolyploid genetics have also circumvented the limitations of primary studies that have relied on software designed for diploid organisms and single-dose markers [157–159].

Given the complexity of the polyploid nature of highbush blueberry (*V. corymbosum*), the first linkage maps were built for interspecific wild diploid populations and were based on only a few individuals and mostly arbitrary PCR-based markers [160–162]. A genetic map was later built using an interspecific wild diploid blueberry population (pseudo-backcross of *V. corymbosum* and *V. darrowii*) [111]. The first genetic linkage map for tetraploid blueberries was an outcrossing F1 population between northern highbush “Draper” and southern highbush “Jewel”. However, the map was built using only hundreds of markers and a diploid approach [146]. A tetraploid map with high marker density and resolution was published by [144], followed by [113], both using southern highbush cultivars as population parents [113, 144]. Collinear syntenic hits were observed between the genetic maps and the “Draper” reference genome [111, 113, 144]. Few differences were observed, suggesting either genotype-specific rearrangements or an artifact of the linkage map construction at those regions. A high degree of collinearity between blueberry and cranberry genetic maps based on a set of 323 common SSR markers have also been observed, indicating the potential for QTL and marker transferability between species [138].

Despite the availability of a few genetic maps for blueberry, a consensus genetic map, based on multiple biparental mapping populations, would be an important resource for the community, allowing more precise linkage disequilibrium analyses as for association with phenotypes and fine mapping of QTLs. A multiparent linkage map is another strategy to overcome the limitations of bi-parental maps. A pangenome for *Vaccinium* will also serve as the foundation of a consensus map.

The objective of QTL mapping analyses is to identify marker-trait associations based on the inheritance of markers within a family-based mapping population. Identifying the genomic regions controlling important horticultural and fruit quality traits is the precursor step for the implementation of marker-assisted selection (MAS). So far, QTL mapping analyses in blueberry have been performed in only three populations: one based on an interspecific cross between wild diploid genotypes [111, 162]; and two based on different southern highbush tetraploid cultivars, one between “Indigocrisp” x “Sweetcrisp” [144] and another between “Reveille” x “Arlen” [113]. Given the early acting inbreeding depression in blueberry, the mapping populations constitute outcrossing-F1 progenies as the segregating generation, one of them followed by pseudo-backcrossing due to interspecific hybridization.

**Figure 2:**
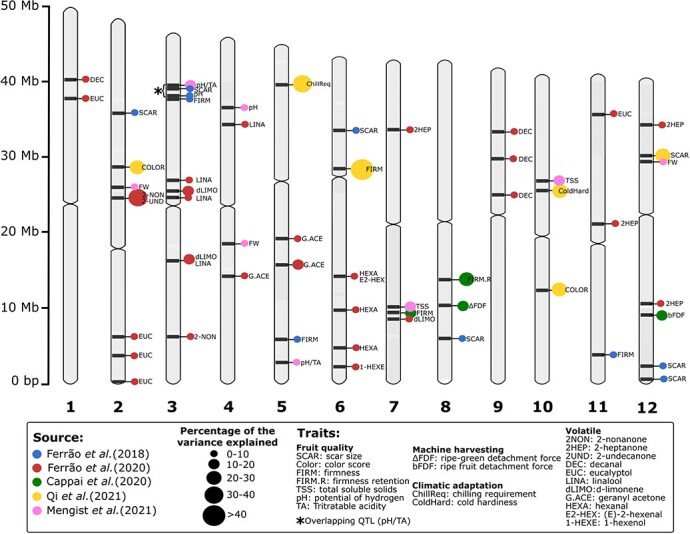
QTLs identified in blueberry through GWAS and QTL mapping studies. Chromosomes represent the largest homologous set of the “Draper” genome [46]. Significant genomic regions from studies using a distinct reference genome were re-assigned using BLAST. Markers on chromosomes represent the central position of the QTL region. The percentage of the phenotypic variance explained by QTLs were estimated based on different methodologies according to the papers referenced in the figure.

To assist in the breeding of cultivars with broader climatic adaptation, the first QTL mapping study focused on understanding the underlying genetics of cold hardiness and chilling requirement related traits [162]. Consistent QTLs were identified for both traits. Seven years later, the same wild diploid population was used in a more comprehensive QTL mapping study, which was improved in terms of marker density, additional individuals, and phenotyping for 18 horticultural traits over multiple years [111] (Table 2). Consistent QTLs were identified for fruit quality, cold hardiness, and chilling requirement (Figure 2).

**Table 2:** Populations, markers, and traits mapped in genotype–phenotype association studies in blueberry

Study	[162]	[110]	[105]	[144]	[111]	[113]
Association	QTL map	GWAS	GWAS	QTL map	QTL map	QTL map
Blueberry type	Wild diploid species	SHB	SHB	SHB	Wild diploid species	SHB
Population	Biparental pseudo backcross	117 full-sib families	92 full-sib families	Biparental outcrossing F1	Biparental pseudo backcross	Biparental outcrossing F1
# Individuals	95	1,559	886	236	117	287
#Markers	265	80,591	71,487	11,292	17,486	17,438
# Traits	2	8	17	5	18	4
Traits	Climatic adaptation	Fruit quality and yield	Flavor-related volatiles	Machine-harvesting related traits	Climatic, fruit quality, developmental	Fruit quality

A tetraploid southern highbush blueberry population was leveraged to map QTLs for machine harvest-related traits, which included: i) high berry firmness; ii) retention of fruit firmness after cold storage; iii) high detachability of ripe fruits; iv) low detachability of unripe/green fruits [144] (Table 2). Two years of evaluations were performed for fruit firmness and only one year of evaluation for the other traits. One consistent QTL was found for fruit firmness across the two years of evaluation [144]. It is noteworthy that firmness-associated QTL from this study did not overlap with the one identified in the wild diploid mapping population [111]. Recently, another tetraploid southern highbush blueberry population was used to map QTLs related to fruit quality (e.g. titratable acidity) [113] (Table 2). Consistent QTLs for at least two years of evaluations were reported for all traits (Figure 2), with the QTL for pH located nearby the one previously identified in a GWAS study [110, 115].

Most QTL mapping studies in crop species have been performed using biparental populations. However, the major drawback of using biparental populations is that few recombination events occur within the population, and QTLs can be located within large chromosomal intervals, making it difficult to narrow down candidate genes and perform positional cloning. Moreover, the limited diversity of the two parents accounts for only a small portion of the genetic variability, reducing the number of alleles and QTL detected and the transferability across populations. To overcome these limitations, multiparent linkage mapping is emerging as a more powerful tool to integrate linkage maps from more diverse genomic backgrounds and joint-family analyses for QTL mapping. Multiparent populations also have the advantage of using populations normally generated in the plant breeding program routine, in contrast to the creation of large experimental biparental populations [163–165]. It is noteworthy that biparental QTL mapping is still a valid approach, especially for mapping rare alleles that may have been underutilized in breeding program history [166].

GWAS, like QTL mapping, is a genomic tool for the identification of genetic variants associated with important traits. However, unlike family-based QTL mapping, GWAS takes advantage of the linkage disequilibrium from historic recombination events accumulated over generations in a diverse panel of genotypes. Thereby, it provides higher resolution for the QTL mapped, makes use of greater allele numbers, detects more frequent QTL in the population, and multiple traits can be assessed. In a cross-pollinating species, such as blueberry and cranberry, it comes at the cost of requiring a higher density of markers throughout the genome and the number of individuals in the association panel. GWAS analyses have been recently performed in southern highbush blueberry for fruit quality and yield-related traits [110, 115], including flavor-related volatile compounds [105] (Table 3). At first, diploid and polyploid methods were compared under a univariate linear mixed model accounting for population and family structure (Q + K model). It was noteworthy that the allele dosage estimation and tetraploid models were important for detecting SNP associations for most traits in contrast to diploidized models [110].

**Table 3:** Populations, markers, and traits mapped in genotype–phenotype association studies in blueberry

**Association**	**Genotype**	**Population**	**Individuals (no.)**	**Markers (no.)**	**Traits (no.)**	**Traits**	**Reference**
QTL map	Wild diploid species	Biparental pseudo backcross	95	265	2	Climatic adaptation	[162]
GWAS	SHB	117 full-sib families	1,559	80,591	8	Fruit quality and yield	[110]
GWAS	SHB	92 full-sib families	886	71,487	17	Flavor-related volatiles	[105]
QTL map	SHB	Biparental outcrossing F1	236	11,292	5	Machine harvesting related traits	[144]
QTL map	Wild diploid species	Biparental pseudo backcross	117	17,486	18	Climatic adaptation, fruit quality, development	[111]
QTL map	SHB	Biparental outcrossing F1	287	17,438	4	Fruit quality	[113]

SHB: Southern highbush blueberry

The GWAS results for fruit quality and yield-related traits (e.g. flower bud density and yield scores) suggested a complex genetic basis. For all these traits, no association or few SNP associations were found explaining a small portion of the observed phenotypic variation (<5%) [110, 115] (Figure 2). Therefore, the following step of marker-assisted selection using the GWAS hits for these traits seems to be limited at this point. Nonetheless, new studies with more individuals, more accurate phenotypes, and higher-quality genomic resources could reveal novel associations.

In another GWAS study to understand the genetic basis of some potential flavor-related volatiles, significant associations were found for 11 compounds [105]. Most of the volatiles were influenced by a few genomic regions, with select individual SNPs explaining >10% of the observed phenotypic variation (Figure 2). Their potential to be levaraged in marker-assisted selection was assessed by using only the GWAS hits to predict the trait in a cross-validation scenario. For some volatiles, the predictive ability performances were similar to the results from genomic selection, where thousands of genome-wide markers were used simultaneously to predict the trait [105]. Biosynthetic enzyme-coding genes were also found within the significant genomic windows for some volatiles, providing plausible candidates for further functional characterization of the causal mutation.

QTL mapping and GWAS studies are paving the way for the use of molecular markers to select superior genotypes. Currently, we are not aware of MAS being fully integrated into any public Vaccinium breeding program; however, recent studies such as the flavor-associated volatiles in blueberry, are very encouraging. Once significant markers explaining a large proportion of the trait variability are identified, MAS can be used to make decisions on parental plants used in crosses, selecting seedlings predicted to have positive traits at early seedling stages, and for pyramiding multiple genes/traits.

The application of MAS is usually recommended for traits with high heritability and are largely qualitative in nature. However, the majority of important target traits are complex and polygenic. In this scenario, genomic selection has been shown to be a more suitable approach. Genomic selection requires high marker density to be simultaneously used to calculate the genomic estimated breeding values (GEBV), therefore, major and small-effect QTLs will be accounted for in the prediction models [167].

Genomic selection is being implemented in select public and private blueberry breeding programs. The lessons learned throughout its implementation have been recently described in detail by Ferrão et al. (2021) [168]. Briefly, the authors have shown that: **i)** genomic selection outperforms pedigree-based predictions [169]; **ii)** simplistic models, such as modeling additive effects under a linear mixed model framework (GBLUP), yielded similar predictive abilities as more complex models [163, 169, 170]; **iii)** regarding genotyping requirements, moderate marker density (around 10 000 SNPs) and low-to-mid sequencing depth (6X–12X) can be used for genomic selection [171]; **iv)** predictive abilities varied across traits in a cross-validation scenario, ranging from 15–51% [169]; **v)** genomic selection is still encouraging after being tested on independent populations [168]; **vi)** preliminary observation of genotype-by-environment interaction warrants further investigation for environment-specific breeding efforts [168].

## Genes and gene editing for high-precision breeding

While traditional breeding through germplasm selection or interspecific hybridization has been widely used for new *Vaccinium* cultivars development [172, 173], polyploidy and heterozygosity often make traditional breeding of *Vaccinium* crops a time-consuming and labor-intensive process. Accordingly, genetic engineering tools could provide a powerful approach for introducing and/or targetting desirable horticultural traits, which would not be possible by traditional breeding [174, 176]. Recent advances in *Vaccinium* genomics and gene editing technologies are stimulating the application of genetic transformation for specific manipulation of the genomes of *Vaccinium* species [44–49, 177]. Genetic engineering relies on the presence of suitable target genes and new biotechnological tools such as efficient transformation protocols and effective gene manipulation [176]. Unlike traditional breeding approaches that enable exchange and recombination of numerous genes, genetic engineering would greatly speed the introduction of individual genes of interest for precision breeding for desirable characteristics of existing cultivars [174]. Great efforts have been made to improve blueberry and cranberry using genetic engineering; however, no genetically modified *Vaccinium* crops have been released for commercialization. This is mainly due to the current high cost of de-regulating them because of the genetically modified organism (GMO)-free policies.

Stable transformation of both blueberry and cranberry has been made possible with the development of the efficient regeneration protocols [178–186]. Genetic transformation of cranberry has, to date, resulted in the development of transgenic cranberry for herbicide resistance [181]. With the efficient transformation and regeneration protocols for blueberry cultivars [182, 183], transgenic blueberries have been developed for herbicide resistance [184, 185], freezing tolerance [71, 186–188], early flowering [77, 189, 190], yield increase [191, 192], and gene knock-out [177]. Using reverse genetics, functional analysis of several blueberry genes has been reported. Freezing tolerant blueberry has been developed by overexpressing a blueberry *DWARF AND DELAYED FLOWER 1* gene [71], and constitutive expression of the blueberry *FLOWERING LOCUS T* has enabled fast-track blueberry breeding through transgrafting [193] Remarkably, manipulating the expression of the Keratin-domain of the blueberry *SUPPRESSOR OF OVEREXPRESSION OF CONSTAN 1* MADS-box gene has provided a high potential to increase blueberry yield as well as grain yield of maize [70, 192]. More interestingly, a transgenic line which overexpresses blueberry *TYPE-B RESPONSE REGULATOR 2* has been identified through whole genome and transcriptome sequencing to study dormancy in blueberries [194, 195]. As a proof-of-concept, these studies have demonstrated that both transgenesis and intragenesis are powerful tools for blueberry improvement. For example, through transgenesis, RNA interference (RNAi) technology will be desirable to overcome the future challenges from the major viruses affecting *Vaccinium* spp. (i.e. red ringspot virus (RRSV), blueberry shoestring virus (BBSSV), blueberry stunt, blueberry scorch virus (BlScV) and blueberry shock virus(BlShV)) [189, 196]. Moreover, comparative transcriptome analysis in transgenic and non-transgenic blueberry plants provides a powerful approach not only to reveal gene functions and gene networks but also to elucidate molecular mechanisms of different pathways (e.g. C-repeat binding factors (CBFs) for cold tolerance, hormone genes and flowering pathway for yield increase) in blueberry [71, 77, 187, 190, 191, 193].

Gene editing using CRISPR/Cas9 can modify an endogenous gene in a precise manner without leaving any footprints and offer an effective solution to alleviating transgenic concerns [197]. This technology has well been demonstrated in many species, including several fruit crops [176]. Development and application of such a genome editing will certainly help improve blueberry cultivars through genomic technologies without transgenic concerns. In fact, the CRISPR/Cas9 system was recently used to knock out blueberry’s *CENTRORADIALIS* gene, where four gRNAs driven respectively by CaMV 35S and Ubi promoters were evaluated in two tetraploid blueberry cultivars and the overall on-target mutation rate was very low [177]. Similarly, when CRISPR-Cas9 and CRISPR-Cas12a were evaluated for their editing efficiencies of a marker gene, *beta-glucuronidase*, only CRISPR-Cas9 led to on-target mutations at low initial editing frequencies (< 5%); unsurprisingly, chimeric mutations were the main problem in production of putatively edited clones (G. Song, Unpublished). These studies have demonstrated that CRISPR Cas9 technologies are powerful tools for facilitating high-precision breeding of blueberries, although substantial effort is still needed to further improve editing efficiency. Currently, a lack of research funds from government and industry sources has slowed the application of genetic engineering for improvement of *Vaccinium* crops. As we obtain more genomic resources for *Vaccinium*, genome engineering will produce cultivars with unique characteristics but without any transgenes that cannot be easily obtained through conventional breeding and will be more easily adopted by the general public.

## Conclusion

The first high-quality chromosome-scale assemblies for blueberry [46], cranberry [48], and bilberry [49] have been published over the past couple of years, which have served as an excellent set of resources for furthering our understanding of the underlying genetics encoding various important target traits. However, future construction of pangenomes for each of the crops will be instrumental to help us further quantify and dissect the diversity present in wild populations and various breeding programs. Efforts to construct pangenomes for blueberry and cranberry, as part of the VacCAP project [53], are currently underway. Several community efforts are also underway to develop new genotyping tools, which when combined with new emerging technologies in phenotyping that are more accurate and high-throughput, will greatly help accelerate genetic discoveries and help advance breeding efforts. This collection of tools and resources will allow us to construct a roadmap to introgress beneficial target gene content from wild germplasm into breeding populations and to guide genetic engineering efforts aimed at developing new superior cultivars. We anticipate that this will be equally impactful for current orphan crops in *Vaccinium* (e.g. ohelo berry), as it has been shown to be a proven strategy in groundcherry (*Physalis pruinosa*) [198]. Lastly, a major area of focus and investment needs to remain on data infrastructure, storage and analyses, and efforts to conserve wild germplasm around the world. A lot of genetic and phenotypic diversity still exists in wild populations, which could be utilized to further improve the resilience of *Vaccinium* crops to diverse (a)biotic stresses and various fruit quality traits. This will remain particularly important as we continue to manage production issues related to global climate change.

## Acknowledgements

This work was supported by Michigan State University AgBioResearch, USDA HATCH 1009804 to P.P.E., USDA HATCH 1018601 to CPL and LMFL, USDA NIFA NRSP 10, and USDA NIFA 2018-67013-27592 and 2019-51181-30015.

## Data availability statement

No data was generated as part of this review paper.

## Conflict of interest

The authors declare no conflict of interest.

## Supplementary data

Supplementary data is available at *Horticulture Research * online.

## Supplementary Material

Web_Material_uhac083
