# Supplementary material for: There and back again; historical perspective and future directions for Vaccinium breeding and research studies
Source: Hortic Res. 2022 Apr 11;9:uhac083. doi: 10.1093/hr/uhac083 (PMC9123236; doi:10.1093/hr/uhac083)
Supplement: Web_Material_uhac083 [file web_material_uhac083.pdf]

Supplemental Table 1. Metabolites identified in different organs of *Vaccinium* spp. at different developmental stages and experimental conditions.

Species	Metabolite class	Organ	Developmental stage	Experimental condition	References
V. corymbosum	Primary metabolites	Fruits	Fruit set		(Montecchiarini et al., 2019)
			Maturity (ripe fruits)		(Kim et al., 2015; Lee et al., 2014; Montecchiarini et al., 2019; Qi et al., 2021)
		Leaves	Vegetative	Aluminum toxicity	(Cárcamo-Fincheira et al., 2021)
		Roots	Vegetative	Aluminum toxicity	(Cárcamo-Fincheira et al., 2021)
	Organic acids	Fruits	Maturity (ripe fruits)		(Wang et al., 2019)
	Secondary metabolites	Fruits	Maturity (ripe fruits)		(Ma et al., 2013; Qi et al., 2021)
	Phenolic compounds	Fruits	Fruit development		(Castrejón et al., 2008; Günther et al., 2020; Li et al., 2019)

For Review Only

		Maturity (ripe fruits)		(Kim et al., 2015; Lee et al., 2014; Mengist et al., 2020)
	Leaves	Vegetative	UV-B treatment	(Inostroza-Blancheteau et al., 2014; Luengo Escobar, Alberdi, et al., 2017; Luengo Escobar, Magnum de Oliveira Silva, et al., 2017)
Flavonoids	Fruits	Fruit development		(Zifkin et al., 2012)
		Maturity (ripe fruits)		(Wang et al., 2019)
Anthocyanins	Fruits	Fruit development		(Günther et al., 2020; Li et al., 2019)
			UV radiation	(Yang et al., 2019)
		Maturity (ripe fruits)		(Lohachoompol et al., 2008)
			Altitude	(Zoratti et al., 2015a)
			Sunlight radiation	(Zoratti et al., 2015b)
Flavonols	Fruits	Fruit development	UV radiation	(Yang et al., 2019)

For Review Only

	Leaves	Vegetative	UV-B	(Luengo Escobar, Alberdi, et al., 2017; Luengo Escobar, Magnum de Oliveira Silva, et al., 2017)
Volatile compounds	Fruits	Maturity (ripe fruits)		(Du & Rouseff, 2014; Ferrão et al., 2020; Polashock et al., 2007; Saftner et al., 2008)
			Location, harvest date	(Du et al., 2011)
			UV-B	(Eichholz et al., 2011)
Triterpenoids, Non-polar compounds	Leaves	Before flowering		(Vrancheva et al., 2021)
Iridoids	Floral buds	Flower bud development		(Leisner et al., 2017)
	Fruits	Maturity (ripe fruits)		(Heffels et al., 2017; Leisner et al., 2017)
	Leaves	Maturity (ripe fruits)		(Leisner et al., 2017)
	Stems	Maturity (ripe fruits)		(Leisner et al., 2017)

For Review Only

V. virgatum	Secondary metabolites	Fruits	Maturity (ripe fruits)	(Ma et al., 2013)
	Polyphenolic compounds	Fruits	Fruit development	(Günther et al., 2020)
	Anthocyanins	Fruits	Fruit development	(Günther et al., 2020)
			Maturity (ripe fruits)	(Lohachoompol et al., 2008)
V. angustifolium	Whole metabolome	Leaves	Reproductive	(Markus et al., 2015)
	Secondary metabolites	Fruits	Maturity (ripe fruits)	(Ma et al., 2013)
	Phenolic compounds	Fruits	Maturity (ripe fruits)	(Harris et al., 2007; Mengist et al., 2020)
		Leaves	Maturity (ripe fruits)	(Harris et al., 2007)
		Roots	Maturity (ripe fruits)	(Harris et al., 2007)
		Stems	Maturity (ripe fruits)	(Harris et al., 2007)
	Iridoids	Fruits	Maturity (ripe fruits)	(Heffels et al., 2017)
V. macrocarpon	Whole metabolome	Leaves	Reproductive	(Markus et al., 2015)

For Review Only

	Polar compounds	Fruits	Maturity (ripe fruits)	(Hurkova et al., 2019)
	Organic acids	Fruits	Maturity (ripe fruits)	(Fong et al., 2020, 2021)
	Phenolic compounds	Fruits	Maturity (ripe fruits)	(Wang et al., 2018)
	Anthocyanins	Fruits	Maturity (ripe fruits)	(Vorsa & Polashock, 2005)
	Non-volatile compounds	Fruits	Maturity (ripe fruits)	(Brown, Murch, et al., 2012; Brown, Turi, et al., 2012)
V. oxycoccos	Anthocyanins	Fruits	Maturity (ripe fruits)	(Vorsa & Polashock, 2005)
	Non-volatile compounds	Fruits	Maturity (ripe fruits)	(Brown, Turi, et al., 2012)
V. vitis-idaea	Polar compounds	Fruits	Maturity (ripe fruits)	(Hurkova et al., 2019)
	Fatty acids	Leaves	Whole growing season	(Liu et al., 2014)
	Phenolic compounds	Leaves	Whole growing season	(Liu et al., 2014)
	Triterpenoids, Non-polar compounds	Leaves	Before flowering	(Vrancheva et al., 2021)

For Review Only

	Non-volatile compounds	Fruits	Maturity (ripe fruits)		(Brown, Turi, et al., 2012)
V. myrtillus	Primary metabolites	Fruits	Fruit development		(Ayaz et al., 2001)
			Maturity (ripe fruits)	Location	(Elisabetta et al., 2013; Rohloff et al., 2015)
				Climate, temperature, photoperiod	(Rohloff et al., 2015; Uleberg et al., 2012)
				Fertilizer, soil	(Rohloff et al., 2015)
	Fatty acids	Leaves	Whole growing season		(Liu et al., 2014)
	Secondary metabolites	Fruits	Maturity (ripe fruits)	Location	(Elisabetta et al., 2013)
	Phenolic compounds	Flowers	Flowering		(Riihinen et al., 2008)
		Fruits	Maturity (ripe fruits)		(Ancillotti et al., 2016; Riihinen et al., 2008)
				Location	(Mikulic-Petkovsek et al., 2015)
				Temperature, photoperiod	(Uleberg et al., 2012)

For Review Only

	Leaves	Whole growing season		(Liu et al., 2014)
		Maturity (ripe fruits)		(Riihinen et al., 2008)
	Rhizome	Maturity (ripe fruits)		(Riihinen et al., 2008)
Flavonoids	Leaves	Maturity (ripe fruits)	Solar radiation	(Jaakola et al., 2004)
	Fruits	Fruit development		(Jaakola et al., 2002)
	Fruits	Maturity (ripe fruits)	Solar radiation	(Jaakola et al., 2004)
Proanthocyanidins	Fruits	Fruit development		(Suvanto et al., 2020)
		Maturity (ripe fruits)	Location	(Åkerström et al., 2010)
Anthocyanins	Fruits	Maturity (ripe fruits)		(Benvenuti et al., 2018; Lätti et al., 2008)
			Location/altitude	(Åkerström et al., 2010; Primetta et al., 2013; Zoratti et al., 2015a)
			Sunlight radiation	(Zoratti et al., 2015b)
Triterpenoids, Non-polar compounds	Leaves	Before flowering		(Vrancheva et al., 2021)

For Review Only

	Iridoids	Fruits	Maturity (ripe fruits)	(Heffels et al., 2017)
V. bracteatum	Primary metabolites	Fruits	Maturity (ripe fruits)	(Lee et al., 2014)
	Secondary metabolites	Fruits	Maturity (ripe fruits)	(Lee et al., 2014)
		Leaves	Post-budding stages	(Fan et al., 2018, 2019, 2020)
V. uliginosum	Phenolic compounds	Fruits	Maturity (ripe fruits)	(Ancillotti et al., 2016)
	Proanthocyanidins, Anthocyanins	Fruits	Maturity (ripe fruits)	(Kellogg et al., 2010)
	Triterpenoids, Non-polar compounds	Leaves	Before flowering	(Vrancheva et al., 2021)
	Iridoids	Fruits	Maturity (ripe fruits)	(Heffels et al., 2017)
V. elliottii	Organic acids	Fruits	Maturity (ripe fruits)	(Yifei Wang et al., 2019)
	Phenolic compounds	Fruits	Maturity (ripe fruits)	(Mengist et al., 2020; Yifei Wang et al., 2019)
	Iridoids	Fruits	Maturity (ripe fruits)	(Leisner et al., 2017)

For Review Only

V. oldhamii	Primary metabolites	Fruits	Maturity (ripe fruits)	(Lee et al., 2014)
	Secondary metabolites	Fruits	Maturity (ripe fruits)	(Lee et al., 2014)
V. ovalifolium	Whole metabolome	Leaves	Reproductive	(Markus et al., 2015)
	Proanthocyanidins, Anthocyanins	Fruits	Maturity (ripe fruits)	(Kellogg et al., 2010)
V. arboreum	Iridoids	Fruits	Maturity (ripe fruits)	(Leisner et al., 2017)
V. arctostaphylos	Primary metabolites	Fruits	Fruit development	(Ayaz et al., 2001)
V. boreale	Organic acids, Flavonoids	Fruits	Maturity (ripe fruits)	(Yifei Wang et al., 2019)
V. calycinium	Iridoids	Fruits	Maturity (ripe fruits)	(Leisner et al., 2017)
V. consanguineum	Iridoids	Fruits	Maturity (ripe fruits)	(Leisner et al., 2017)
V. corymbodendron	Iridoids	Fruits	Maturity (ripe fruits)	(Leisner et al., 2017)
V. cylindraceum	Iridoids	Fruits	Maturity (ripe fruits)	(Leisner et al., 2017)
V. darrowii	Organic acids, Flavonoids	Fruits	Maturity (ripe fruits)	(Yifei Wang et al., 2019)

For Review Only

V. floribundum	Iridoids	Fruits	Maturity (ripe fruits)	(Leisner et al., 2017)
V. fuscatum	Iridoids	Fruits	Maturity (ripe fruits)	(Leisner et al., 2017)
V. ovatum	Iridoids	Fruits	Maturity (ripe fruits)	(Leisner et al., 2017)
V. padifolium	Iridoids	Fruits	Maturity (ripe fruits)	(Leisner et al., 2017)
V. pallidum	Organic acids, Flavonoids	Fruits	Maturity (ripe fruits)	(Yifei Wang et al., 2019)
V. reticulatum	Iridoids	Fruits	Maturity (ripe fruits)	(Leisner et al., 2017)
V. stamineum	Iridoids	Fruits	Maturity (ripe fruits)	(Leisner et al., 2017)
V. tenellum	Organic acids, Flavonoids	Fruits	Maturity (ripe fruits)	(Yifei Wang et al., 2019)

For Review Only
